# Squamous cell carcinoma of tongue in HIV patients- a report of 3 cases

**DOI:** 10.1186/1471-2334-14-S3-E38

**Published:** 2014-05-27

**Authors:** SP Selvajothi Ranjitham, K Gnanambigai, P Kamaleswari, Elizabeth Joshua, Umadevi K Rao, K Ranganathan

**Affiliations:** 1 Department of Oral Pathology and Microbiology, Ragas Dental College, Chennai, India

## Case series

Oral manifestations of HIV disease are broadly classified as “strongly associated with HIV”, “less commonly associated with HIV” and “can be seen in HIV infection”. Malignancies reported in HIV infection include Kaposi’s sarcoma, Non-Hodgkin’s lymphoma and squamous cell carcinoma of the oral cavity. At our Ragas- YRG CARE center, in a cohort of 5760 cases which were screened, 6 patients of Non-Hodgkin’s lymphoma and 4 patients with oral squamous cell carcinoma were reported. We present 3 cases of oral squamous cell carcinoma on the lateral border of the tongue with varied clinical presentations. A comparison of the demographic data, habits, HIV history, clinical presentation, investigations, histopathological diagnosis and treatment of the three cases were made; 2 patients had no history of tobacco use, suggesting role of factors such as HPV co infection.

